# Control of crystallization behaviour of supercooled liquid composed of lithium disilicate on platinum substrate

**DOI:** 10.1038/s41598-017-06306-9

**Published:** 2017-07-20

**Authors:** Masanori Tashiro, Sohei Sukenaga, Hiroyuki Shibata

**Affiliations:** Institute of Multidisciplinary Research for Advanced Materials, Tohoku University, Katahira 2-1-1, Aobaku, Sendai 980-8577 Japan

## Abstract

Crystalline lithium disilicate (Li_2_Si_2_O_5_, LS2) materials, which have excellent mechanical properties with high transparency, should be obtained efficiently through the crystallization of supercooled liquid composed of LS2. However, in addition to LS2, a lithium monosilicate (Li_2_SiO_3_, LS) phase is also precipitated during the crystallization of the liquid. The precipitation of the LS phase renders it difficult to obtain a single-phase LS2 material. Here, we show that by altering the oxygen partial pressure, it is possible to change the selectivity of the precipitated phase by controlling the interfacial phenomena that occur between the liquid and platinum contact material. During cooling of the supercooled liquid, the type of precipitated phase can be controlled by optimizing the atmosphere and type of contact material. This methodology can be applied for the fabrication of other functional materials and does not require the use of other additives.

## Introduction

Silicate crystal has many functions depending on the local structure of its silicon atoms, which is commonly classified by the Q^*n*^ species, where *n* represents the number of bridging oxygen atoms around a SiO_4_ tetrahedron. Generally, an increase in the *n* value increases the physical and chemical toughness of the material, while silicate crystals with a lower *n* value (<2) can be highly reactive (e.g. CO_2_ capture materials^1^). Lithium disilicate (Li_2_Si_2_O_5_, LS_2_) crystals are a good example of Q^3^-based silicate crystals with excellent chemical and physical durability^2, 3^. LS2 crystals (melting point: 1306 K), which can be obtained by crystallization of their supercooled liquids^4^, have recently been applied as dental materials. Recent simulations^5^ estimate that LS2 potentially has a band-gap of 5–7 eV; this band gap may lie between the band gap ranges of semiconductors and insulators. This scenario shows that single crystals of LS2 can be applied to functional materials as well as structural materials. The crystallization behaviour of supercooled liquids composed of LS2 has been studied for many years^6^. A recent thermodynamic calculation^7^ showed that a LS2 phase alone should be formed from liquid composed of LS2, but experimental data^6^ show that a lithium monosilicate (Li_2_SiO_3_, LS) phase will also form from this liquid. Zanotto and co-workers proposed^8^ that the LS phase, which is composed of a Q^*2*^ chain, is more readily nucleated than the LS2 phase because the Q^*2*^ species may have greater mobility than the Q^*3*^ species. Therefore, it was difficult to obtain the LS2 phase without the precipitation of LS or solid-state phase transformation from LS to LS2. Previously, additive oxides (e.g. Al_2_O_3_, P_2_O_5_)^9–18^ were used to control crystallization behaviour of lithium silicate liquids, which rendered it difficult to produce high-purity single-phase LS2 materials. Moreover, during previous studies, supercooled liquids that did not contain such additives were cooled at a relatively high cooling rate (>10 K/min); the results showed that the measured crystallization temperatures were widely scattered and varied by more than 100 K, even though the experimental conditions were kept constant^19–21^. Platinum is considered one of the least reactive materials with respect to oxide liquid and hence it is one of the most commonly used contact materials for oxide liquids. However, the wettability of oxide liquid on platinum is strongly dependent on the atmosphere^22^. Indeed, platinum is only slightly soluble when in contact with silicate melts; however, platinum ions can influence the crystallization behaviour of supercooled silicate liquids^23, 24^. Farges *et al*.^25^ demonstrated that platinum cations within silicate melts are surrounded by several modifier cations (e.g. lithium). Assuming platinum is partially ionized near the interface between the silicate melts and solid platinum, lithium cations would become concentrated near the interface. Such microsegregation of the lithium cations may have an influence on the crystallization behaviour of supercooled LS2 liquids. Here, we investigate the crystallization behaviour of a supercooled liquid composed of LS2 on a platinum substrate under various oxygen partial pressures.

To simultaneously evaluate the drop shape and crystallization behaviour, a gas-tight furnace with customized apparatus^26^ was used. This apparatus allowed us to observe the liquid shape and crystallization behaviour in both vertical and horizontal directions (see Supplementary Figure S1). The glassy samples composed of LS2 with very small amount of platinum (4 ppm, see Supplementary Table S1) were heated to 1473 K on a platinum substrate under Ar and dry-air atmospheres for complete melting to occur. Subsequently, contact angle measurements were performed at 1473 K for 30 min. Using a cooling rate of 20 K/min, the crystallization behaviour of the liquid on the platinum substrate was observed under Ar and dry-air atmospheres.

## Results and Discussion

**Figure 1:**
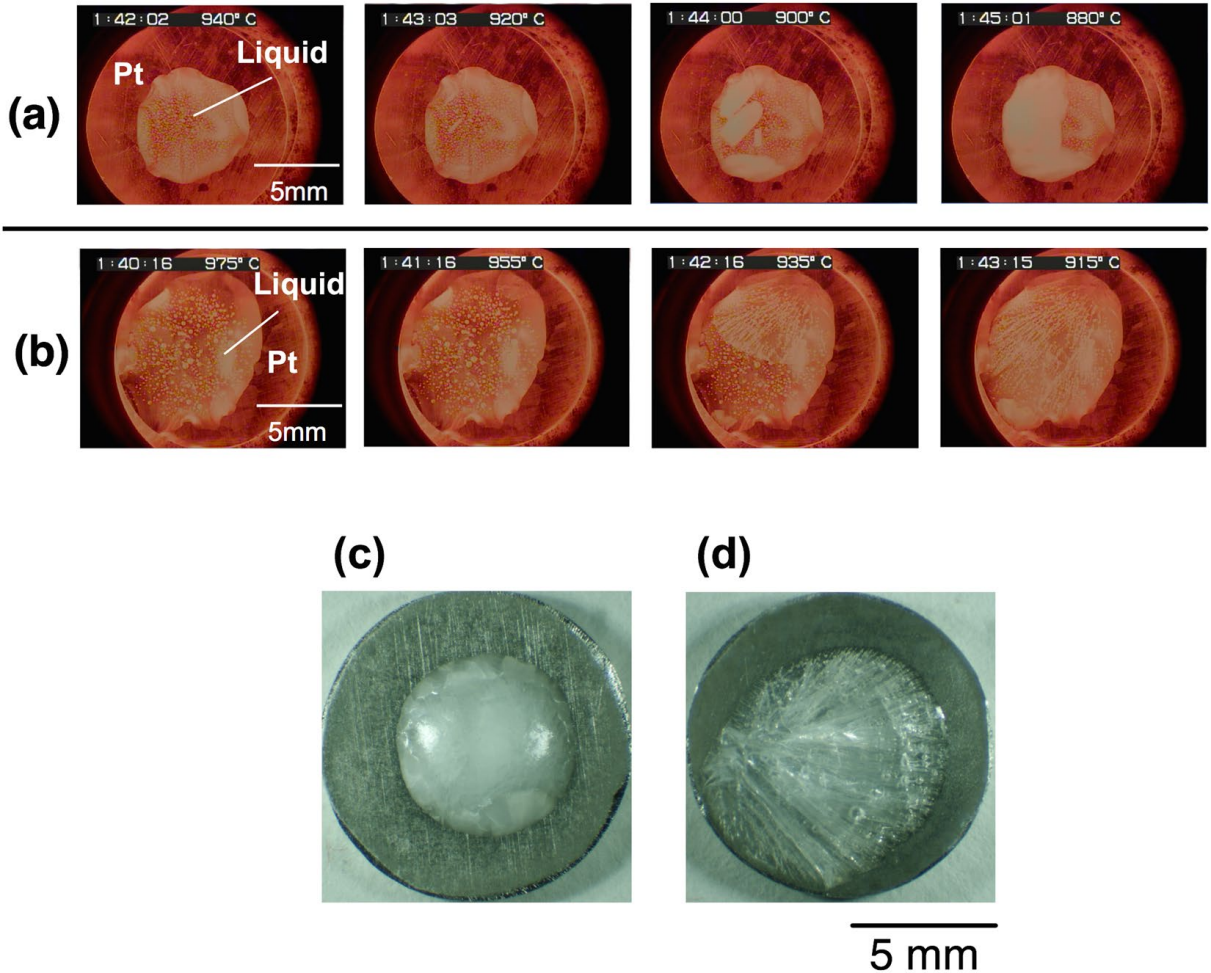
Crystallization behavior (top view) of supercooled liquid composed of lithium disilicate cooled from 1473 K at 20 K/min under (**a**) Ar (*P*_O2_ ≈ 10^−5^ atm) as an example of Type Ι crystallization, and (**b**) under dry–air (*P*_O2_ = 0.2 atm) as an example of Type ΙΙ crystallization. The temperature values on the images represent the temperatures measured using a thermocouple located near the substrate. Images of cooled samples (**c**) under Ar atmosphere with Type Ι crystallization, and (**d**) under dry–air with Type ΙΙ crystallization.

**Figure 2:**
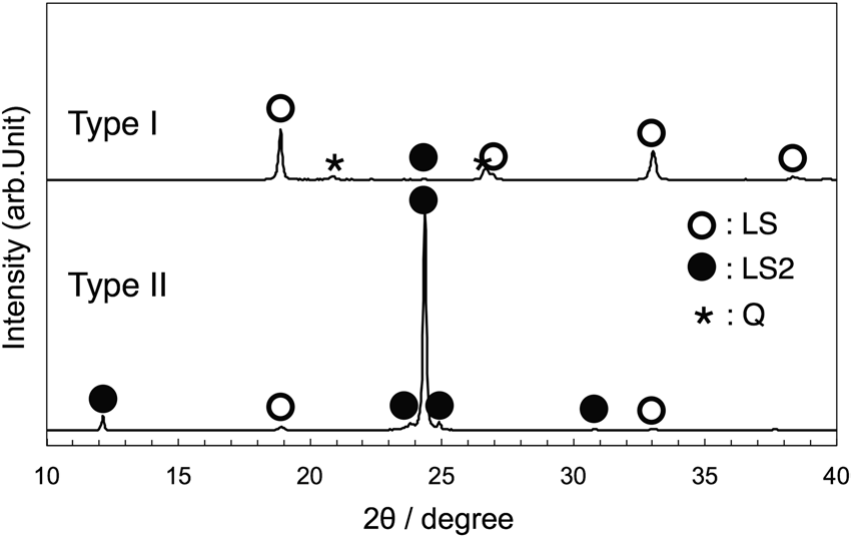
X-ray diffraction patterns of cooled samples under Ar, with Type Ι crystallization, and dry–air, with Type ΙΙ crystallization.

**Figure 3:**
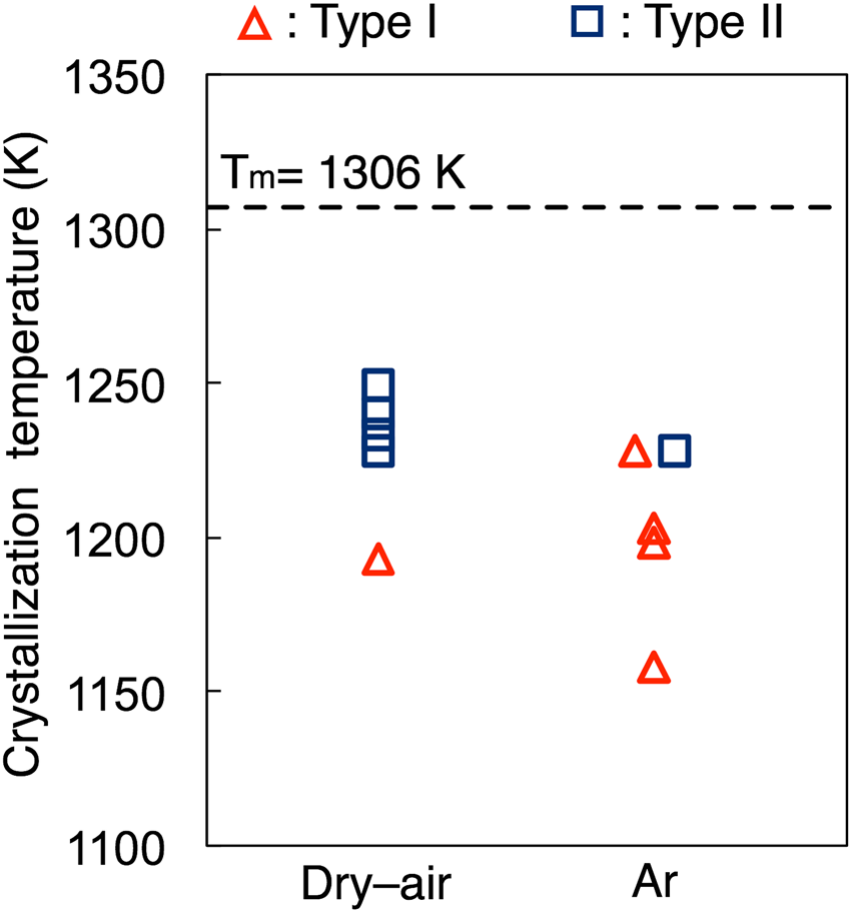
Crystallization temperature and crystallization type of the supercooled liquid composed of lithium disilicate cooled from 1473 K at 20 K/min under Ar (*P*_O2_ ≈ 10^−5^ atm) and dry–air (*P*_O2_ = 0.2 atm) atmospheres. The dashed line represents the melting temperature of the lithium disilicate phase^8^.

Figure 1(a) and (b) show the crystallization behaviour, observed in the vertical direction, of the supercooled liquid under various oxygen partial pressures (*P*_O2_). Figure 1(a) shows the typical crystallization behaviour under an Ar atmosphere (*P*_O2_ ≈ 10^−5^ atm); during cooling, white crystals were primarily precipitated and the growth of the crystals was accompanied by the precipitation of semi-transparent crystals (Type Ι crystallization). Figure 1(b) shows typical crystallization behaviour under a dry-air atmosphere, which differs to that of Type Ι; crystal growth of the semi-transparent phase commenced from the periphery of the droplet and just a small quantity of white crystals formed (Type ΙΙ crystallization). Figure 1(c) and (d) show photographs of the cooled samples following Type Ι (opaque, under Ar atmosphere) and ΙΙ (semi-transparent, under dry-air atmosphere) crystallization, respectively. To evaluate the types of crystalline phases observed following Type Ι and ΙΙ crystallization, X-ray diffraction (XRD) analysis was performed (see Fig. 2). Following Type I crystallization under an Ar atmosphere, the XRD patterns show that the crystallized sample primarily consists of LS, and coexists with LS2 and a small quantity of quartz (SiO_2_, Q). Furthermore, following Type II crystallization, the primary phase of the sample consists of LS2 with a small amount of LS. Therefore, based on the top-view observation and XRD results, the semi–transparent and white phases are determined to be LS2 and LS, respectively. The crystallization behaviour was observed five times under each type of atmosphere. The crystallization temperature was defined as the temperature at which the primary phase was precipitated. Figure 3 shows the observed crystallization types plotted as a function of the crystallization temperature. Type Ι crystallization primarily occurs under an Ar atmosphere, while Type ΙΙ crystallization is more pronounced under a dry-air atmosphere. The crystallization temperature varies widely (>50 K), which corresponds with results of previous studies that were obtained by differential scanning calorimetry under a similar cooling rate and atmosphere^21^. A theoretical phase diagram indicates that a supercooled liquid composed of LS2 should form a LS2 phase only^7^. However, it was clear that both LS2 and LS phases precipitated from the liquid. Furthermore, it was determined that the crystallization behaviour of the liquid strongly depends on the atmosphere.

**Figure 4:**
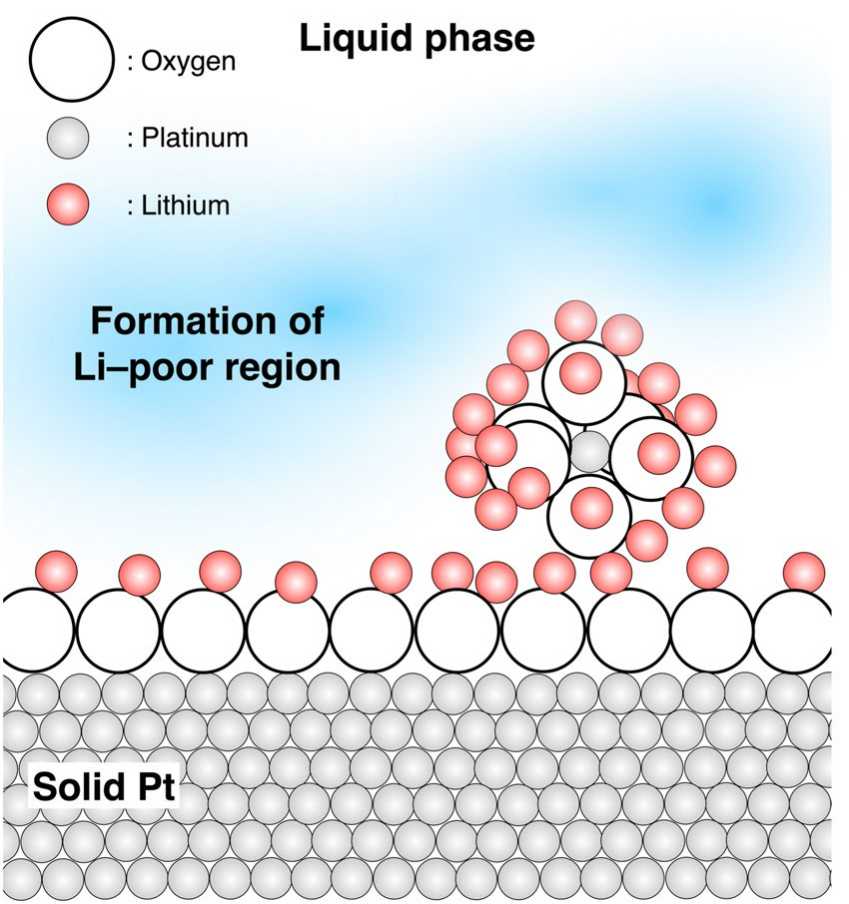
Schematic of local structure of platinum atoms in the supercooled liquid composed of lithium disilicate. It is assumed that the platinum atoms near the liquid/solid interface are partly ionized as Pt^4+^. Lithium cations are segregated in the vicinity of the platinum atoms. Subsequently, a lithium-poor region forms.

**Figure 5:**
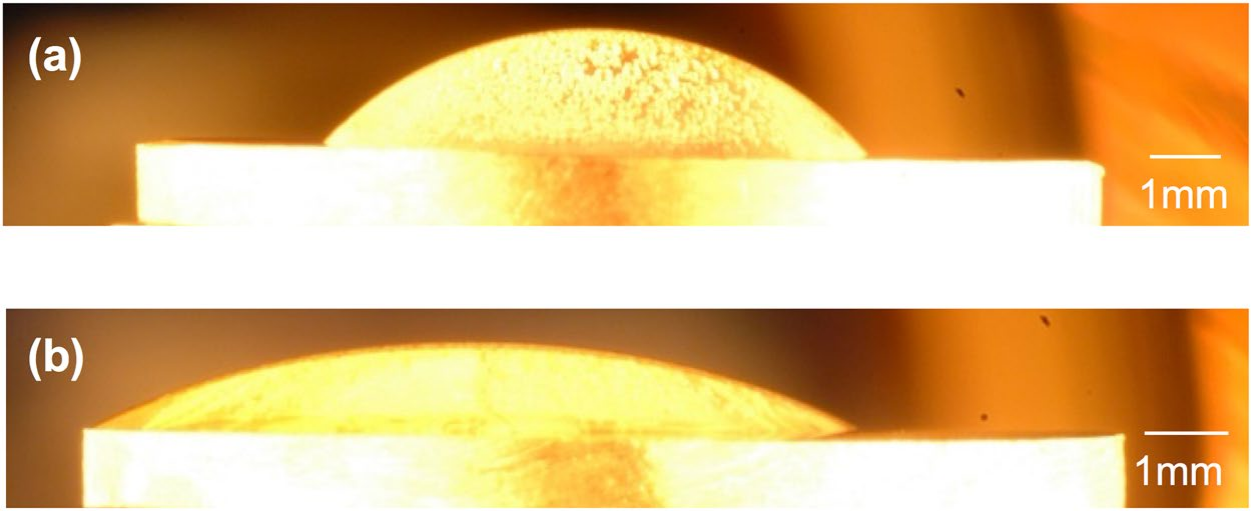
Horizontal view of drop shapes on platinum substrate at 1473 K under (**a**) Ar and (**b**) dry–air atmospheres.

To determine the extent of the reaction between the liquid and platinum, the platinum concentration of the quenched glassy samples was measured via wet chemical analysis under two different atmospheres. Under Ar and dry-air atmospheres, the platinum concentrations were 4 ppm (*P*_O2_ ≒ 10^−5^ atm) and 19 ppm (*P*_O2_ = 0.2 atm), respectively (also see Supplementary Table S1). These values almost correspond to the reported platinum solubility at 1723 K in lithium silicate melts of a similar composition^27, 28^. Researchers have previously discussed the valence state of platinum ions in silicate glasses and melts^25, 28, 29^. The relationship between the platinum solubility and oxygen partial pressure indirectly suggests that platinum cations may present as divalent (Pt^2+^) or hexavalent (Pt^6+^) cations. Furthermore, direct observations during X-ray absorption fine structure (XAFS) experiments^25^ indicated that the platinum valence in silicate should be primarily tetravalent (Pt^4+^) and the cations were in six-fold coordination with oxygen atoms^25^. In addition, network modifiers (e.g. Li^+^) tend to offset the negative charges of the oxygen atoms that surround the platinum cations, while network formers, i.e. silicon cations, do not tend to exist in the vicinity of the platinum cations. Assuming that the platinum atoms near the platinum/liquid interface are partly ionized as Pt^4+^ cations, lithium cations would segregate around the platinum cations in the bulk material and liquid/solid interface. This interfacial phenomenon is schematically described in Fig. 4. The contact angle of the liquid on the platinum substrate decreased as the oxygen partial pressure increased, indicating that the size of the interfacial area increases as the oxygen partial pressure increases, as described in Fig. 5. Recent *in-situ* Raman experiments^30^ have shown that the local structure of silicon atoms in LS2 melts can be approximately described as 20%Q^2^–60%Q^3^–20%Q^4^. Since lithium cations would be trapped in the vicinity of the platinum cations, a lithium-poor region would develop near the liquid/solid interface. In that region, a proportion of the Q^2^ species would transform into Q^3^ species because of the lithium deficiency. Therefore, the microsegregation of lithium in the vicinity of the platinum cations should increase the Q^3^/Q^2^ ratio in that region. This phenomenon would suppress the nucleation of LS crystals, which consist of Q^2^ species^8^, and would promote precipitation of the LS2 phase. Type ΙΙ crystallization preferentially occurs under a dry-air atmosphere with larger platinum/liquid interfacial area because a greater quantity of lithium cations are trapped in the vicinity of the platinum cations compared with that under Ar conditions. Furthermore, under lower oxygen partial pressures resulting from the Ar atmosphere, the level of platinum ionization decreased near the liquid/solid interface. This resulted in the preferential formation of the LS phase, which consisted of Q^2^ units with greater mobility than that of Q^3^ units.

Here, we found that the phase precipitated from the supercooled LS2 liquid could be selected and controlled by altering the oxygen partial pressure or selecting the type of contact material. Our finding offers the potential for high-purity LS2 crystals to be fabricated without the addition of other oxides (e.g. P_2_O_5_). In addition, our methodology can be applied to the fabrication of other functional glass–ceramic materials from supercooled oxide liquids.

## Method

The oxide samples were prepared from reagent grade Li_2_CO_3_ (≧99.0%) and SiO_2_ (99.9%) powders. Specific quantities of the reagent powders were accurately weighed and thoroughly mixed to form melts with target compositions. The powder mixture was placed in a platinum crucible, calcined at 1073 K for 1 h, and subsequently melted at 1673 K in air for 20 min using a resistance furnace. Subsequently, the melt was quenched on a copper plate and poured into a copper mould to obtain glass rod samples with a diameter of 5 mm. The obtained rod-shaped samples were held at an annealing temperature of 713 K for 3 h and subsequently cooled to 298 K. The glass rods were cut and polished into circular shapes with a thickness and diameter of 1.1 mm and 5 mm, respectively (starting material). This starting material has only 4 ppm platinum (see Supplementary Table S1). Since the melting time was short and liquid quantity (15 g) was large, the platinum concentration is much lower than platinum solubility (>15 ppm^27, 28^) under air at 1673 K. Platinum plates (99.95% purity) were used as solid substrates, which were cut into circular shapes with a thickness and diameter of 1 mm and 11 mm, respectively. The substrates were polished using SiC papers, and then ultrasonically cleaned in acetone.

We used the sessile drop method to simultaneously observe the drop shape and crystallization behaviour. Figure S1 shows a schematic of the apparatus used. The glass rods were placed on a platinum substrate, which was placed on a sapphire plate on a platinum plate, located on an alumina tube pedestal. The temperature was measured using a thermocouple that was welded to the base of the platinum plate. Quartz plates were used to separate halogen lamps from the sample chamber. Subsequently, using two halogen lamps, the sample was heated at 20 K/min through the quartz plates. The sample was maintained at a target temperature of 1473 K for 30 min. The drop shape (i.e. contact angle) of the molten oxide on the substrate was measured over a period of 30 min. Through a side window, a digital camera was used to capture images of the horizontal view of the sample at 10 min intervals. Subsequently, the samples were cooled at 20 K/min. Using a CCD camera, the crystallization behaviour of the samples was monitored through the top window during the cooling process. The measured temperatures were superimposed on a video, which was used to record the experimental work. When the sample was cooled to 298 K, the crystallized samples were identified using X-ray diffraction analysis (XRD) with CuKα radiation, a 2θ angle range of 10–70°, scanning rate of 2°/min, and interval of 0.02° (2θ). The phases were identified by comparing the experimental X-ray diffraction patterns to standard patterns compiled by the International Centre for Diffraction Data (ICDD).

Furthermore, to confirm the concentrations of lithium and platinum in the oxide samples, the starting materials on the platinum substrate were heated to 1473 K at a rate of 20 K/min. Subsequently, under both atmospheres, the samples were melted at 1473 K over 30 min and were quenched by deactivating the furnace. The chemical composition of the quenched glassy samples was determined using wet chemical analysis. The lithium concentration of the samples was determined using atomic absorption spectrometry (AAS). The platinum content of the samples before and after experiments was determined using inductively coupled plasma-mass spectrometry (ICP-MAS). The lithium and platinum concentrations are listed in Supplementary Table S1. The lithium concentration remained constant throughout the experiments.

### Data Availability

The datasets generated during and/or analysed during the current study are available from the corresponding author on reasonable request.

## Electronic supplementary material

Supplementary information
