# Supplementary material for: Control of crystallization behaviour of supercooled liquid composed of lithium disilicate on platinum substrate
Source: Sci Rep. 2017 Jul 20;7:6078. doi: 10.1038/s41598-017-06306-9 (PMC5519763; doi:10.1038/s41598-017-06306-9)
Supplement: Supplementary file 1 — Supplementary information [file 41598_2017_6306_MOESM1_ESM.pdf]

Supplementary information

Control of crystallization behaviour of supercooled liquid composed of lithium disilicate on platinum substrate

Masanori TASHIRO, Sohei SUKENAGA*, Hiroyuki SHIBATA

Institute of Multidisciplinary Research for Advanced Materials (IMRAM), Tohoku University, Katahira 2-1-1, Aobaku, Sendai, 980-8577 Japan.

*E-mail address of corresponding author: sukenaga@tagen.tohoku.ac.jp

Supplementary Table S1 | Lithium oxide (mol%) and platinum (mass ppm) concentrations of glassy samples recorded prior to and following melting on platinum substrates at 1473 K for 30 min under two types of atmospheres.

	Li_2O concentration (mol%)	Pt concentration (ppm)
Starting Material (Initial)	32.2	4
After experiment (Dry-air)	32.7	19
After experiment (Ar)	31.4	4

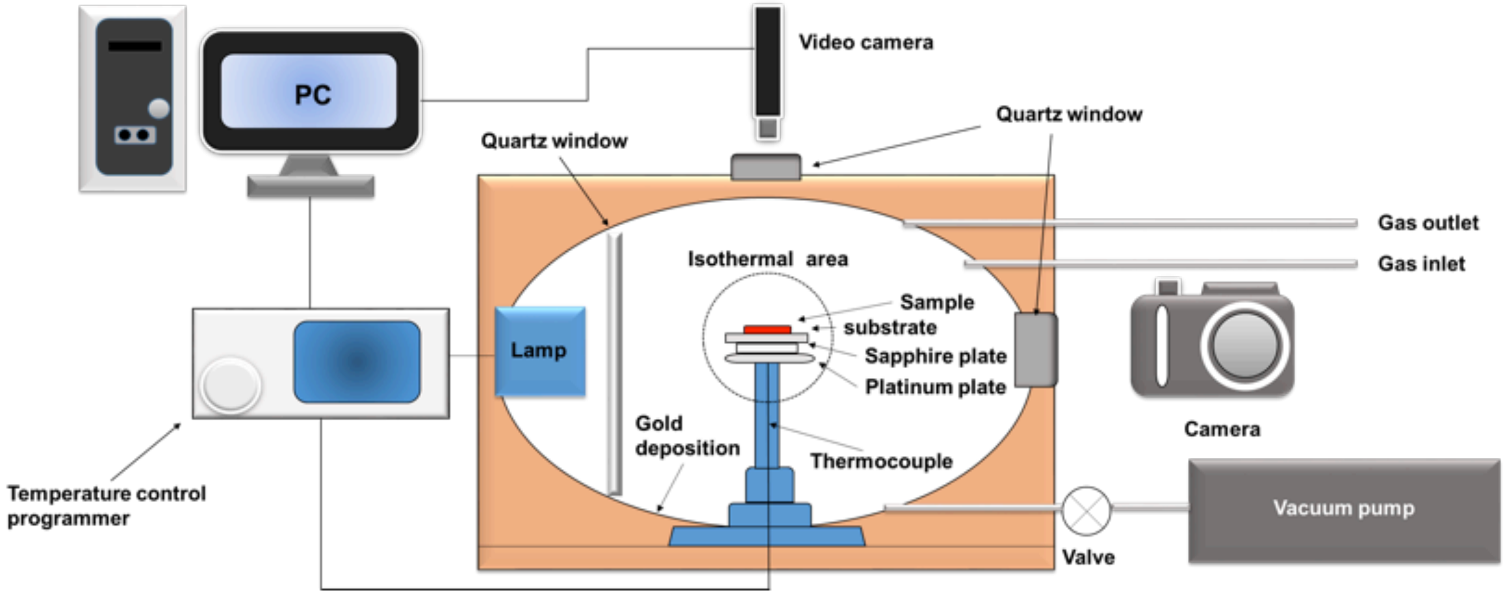

Supplementary Figure S1 | Schematic of apparatus used to observe crystallization behaviour and drop shape.
